# Nonpharmacologic Management of Recurrent Painful Ophthalmoplegic Neuropathy: A Case Report

**DOI:** 10.7759/cureus.84387

**Published:** 2025-05-19

**Authors:** Tyler D Kemp, Robert J Trager

**Affiliations:** 1 Chiropractic Medicine, Private Practice, Middletown, USA; 2 Chiropractic Medicine, Connor Whole Health, University Hospitals Cleveland Medical Center, Cleveland, USA

**Keywords:** acupuncture, chiropractic, migraine, neuropathy, spinal manipulation

## Abstract

Recurrent painful ophthalmoplegic neuropathy (RPON) is a rare neurologic disorder characterized by headaches and ocular cranial nerve paresis with no consensus on optimal treatment. While pharmacologic treatments are commonly used, limited research has explored nonpharmacologic options. A 36-year-old woman with a multi-year history of episodes of RPON lasting several months presented to a chiropractor with a week-long episode of headache and diplopia unresponsive to oral steroids and sumatriptan. Spinal manipulation and acupuncture were administered over six weeks, leading to complete symptom resolution. This is a case of RPON managed successfully with chiropractic care and acupuncture, suggesting a potential beneficial role of these therapies. Given the limitations of a single case, further research is warranted to determine the effectiveness of these therapies for RPON.

## Introduction

Recurrent painful ophthalmoplegic neuropathy (RPON), previously referred to as ophthalmoplegic migraine, is a rare condition characterized by recurrent unilateral headaches followed by ipsilateral ocular cranial nerve paresis, most commonly involving cranial nerve (CN) III. RPON is rare, with an estimated incidence of 0.7 per million person-years [1-2]. The International Classification of Headache Disorders (ICHD-3) defines RPON as requiring at least two attacks, with exclusion of structural lesions via imaging and headaches not meeting criteria for other diagnoses [3]. Its pathophysiology and etiology remain uncertain, with potential triggers including nerve compression, ischemia, inflammation, demyelination, or migraine-related mechanisms [4].

RPON exhibits a range of clinical features. The mean age of onset is 22 years yet ranges widely from early childhood to older adulthood [3]. There is a slight female predominance (i.e., 1.4:1 female-to-male ratio) [4]. Presenting symptoms include unilateral headache with orbital, peri-orbital, supra-orbital, or retro-orbital pain [3,5] followed by paresis of one or more CNs, with a range of other potential symptoms such as nausea, vomiting, phonophobia, and/or photophobia. RPON-related headaches may precede ocular symptoms by up to two weeks [6] and vary in duration from days to several months, while ophthalmoplegia typically lingers from weeks to months [2].

Diagnostic criteria for RPON per the ICHD require at least two attacks, including a unilateral headache and ipsilateral paresis of one, two, or all three ocular motor nerves. Additionally, orbital, parasellar, or posterior fossa lesions must be excluded by appropriate investigations (e.g., imaging), and the condition must not be better accounted for by another headache diagnosis [7]. Alternatively, Liu et al. proposed a modified diagnostic criterion that included additional features, specifically, clinical signs of paresis of CN III, IV, or VI on magnetic resonance imaging (MRI) with ipsilateral ophthalmoplegia with headache onset of less than 15 days [3].

Several case reports have suggested that glucocorticoids may alleviate symptoms of RPON [3,7,8]. However, the efficacy of glucocorticoids has yet to be rigorously tested for RPON in larger study designs, and therefore, their effectiveness and safety remain unclear [4]. Additionally, other medications have been used for RPON, such as nonsteroidal anti-inflammatory drugs and triptans. In general, there remains a lack of consensus regarding the optimal treatment strategies due to the body of literature consisting of case reports. Additionally, there are few alternative medications for recurrent episodes, and no nonpharmacological treatments have been described for RPON in the existing literature.

## Case presentation

A 36-year-old female schoolteacher presented to a private chiropractic clinic in July 2024 with a one-week history of left-sided orbital headache rated 8/10 on the Visual Analog Scale and associated diplopia and light sensitivity. She also noted that tilting her head to the right side reduced her diplopia symptoms. She reported a multi-year history of annual episodes lasting six to nine months with gradual resolution per episode. Her first episode of these symptoms was in 2019. Her most recent episode occurred the year prior and lasted approximately nine months. During past episodes, she received multi-month, low-dose oral steroids with moderate improvement at the third month.

**Figure 1:**
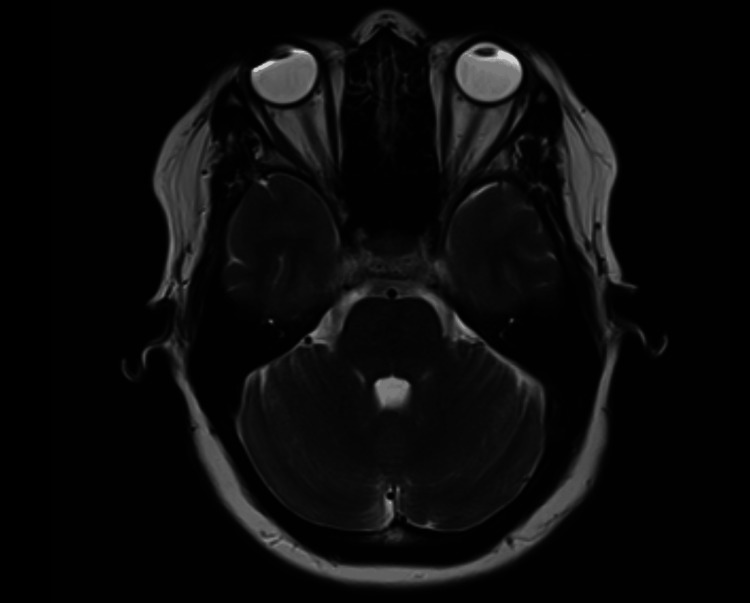
Magnetic resonance image of the brain. An illustrative axial T2-weighted section is shown, with no abnormalities detected in this section or others

For the current headache episode, she initially presented to a major medical center for neurologic evaluation. Brain MRI excluded lesions or nerve effacement (Figure 1). A neurologist confirmed the diagnosis of RPON and prescribed a 10-day course of oral prednisone (20 mg) along with daily sumatriptan (100 mg), which yielded no improvement. As the patient had a previous positive experience with chiropractic care for mechanical back pain and had limited success with medical management for RPON, she sought chiropractic care for her current headache episode. She expressed frustration about the duration of RPON episodes and wished to "shorten the time".

Her past medical history was negative for metabolic disease, and she had no family history of neurologic disorders or migraines and had never smoked. She had no other relevant past medical history.

**Figure 2:**
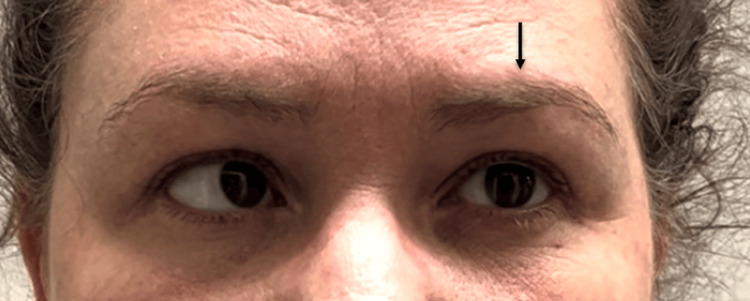
Attempting leftward gaze. The left eye remains fixed in neutral due to left cranial nerve VI (abducens) palsy, while the right eye fully adducts. Image obtained and shown with patient consent. Arrow added for emphasis

Neurological examination revealed 2+ muscle stretch reflexes bilaterally, negative Hoffman's sign bilaterally, intact gait, and no difficulty with Romberg's test or tandem gait. Examination of CN II-XII was unremarkable with the exception of CN VI; upon horizontal visual field testing, the patient was unable to abduct her left eye (Figure 2). Cervical spine range of motion was limited in left rotation and extension and right lateral flexion. Spinal examination revealed reduced segmental joint motion at C1, C5, and T3 via manual palpation, with tenderness.

**Figure 3:**
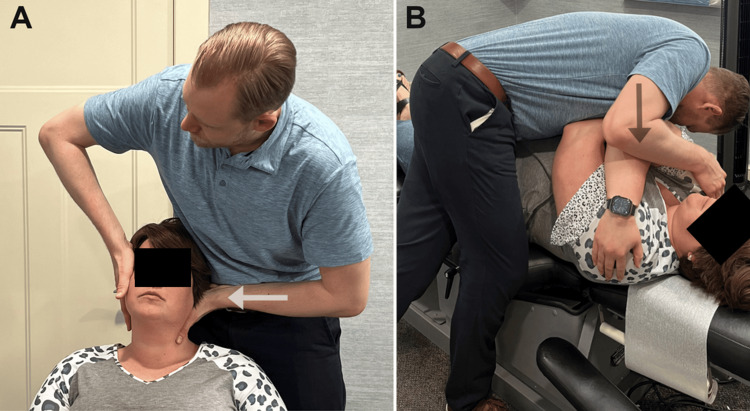
Spinal manipulation Image A depicts a re-enactment of the manipulation used in the present case, with the subject seated. The clinician places their hand at the posterolateral upper cervical spine, in this case at C1 on the left, with the contralateral hand acting to stabilize the head. An anteromedial high-velocity low-amplitude manipulative thrust is then delivered (white arrow). Image B depicts a re-enactment of supine thoracic manipulation, with the clinician's hand placed in the upper and mid-thoracic region and a thrust delivered using downward pressure from the clinician's body (black arrow).

**Figure 4:**
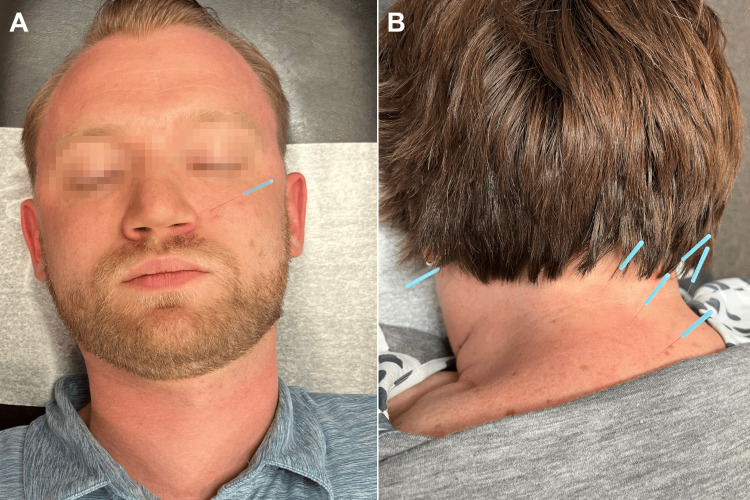
Acupuncture therapy Examples of key head/neck acupoints used in the current case are shown. Needles shown are 40×0.25 mm with light blue handles. Image A depicts a re-enactment of the Stomach 2 point on the left side of the face. Image B depicts the Gallbladder 2 point at the left mastoid process and the Huatuojiaji C2-5 points in the posterior cervical spinal region, lateral to the spinous processes of the C2 through C5 vertebral levels.

The patient consented to a trial of care consisting of six visits at three times per week for two weeks. High-velocity low-amplitude spinal manipulation was performed on the areas of restriction identified in the cervical spine and thoracic spine in both supine and seated positions (Figure 3). Due to associated neurological features, acupuncture was used, targeting the acupoints Huatuojiaji (HJJ) C2-5, Large Intestine (LI) 4, and Lung (LU) 10 for 10 minutes in a prone position. Points were located at the posterior cervical spine, hand, and forearm, respectively, and were selected per traditional Chinese medicine (TCM) principles (Figure 4). At the third visit, the patient reported improvement with complete resolution of her headache and reduced bouts of diplopia. No change was noted prior to that visit.

Care was continued at the frequency of twice per week for three weeks, with the addition of acupoints to now include Stomach (ST) 2 and Gallbladder (GB) 12. At the 12th visit, the patient noted complete abolishment of light sensitivity and diplopia. On exam, cervical range of motion was full and pain-free, and the CN VI was intact bilaterally with no symptoms in the horizontal field. At the one-month follow-up, the patient remained at resolution and was pleased with the abolishment of the complaint in under six weeks. All treatments were tolerated without incident.

In February 2025, the patient presented again with a new episode of RPON with focal headache and diplopia. Neurological testing was unremarkable, with the exception of CN VI, as the patient was unable to abduct her left eye. Her health history was unchanged since the prior episode seven months earlier. The only medication change was the addition of Cymbalta (30 mg) in January 2025. Another trial of care began consisting of the same treatment as the last episode (spinal manipulation to the cervical and thoracic spine and acupuncture). In this episode, the patient experienced full resolution of diplopia and headache at the seventh visit. The total duration of care for this episode was less than six weeks. Again, the RPON episode resolved within six weeks, while prior episodes preceding spinal manipulation and acupuncture had lasted six to nine months.

## Discussion

This case is the first to document successful nonpharmacologic management of RPON episodes using chiropractic care and acupuncture after a lack of response to other common medications, including glucocorticoids. A PubMed literature search performed in April 2025 with the included terms "chiropractic" and "recurrent painful ophthalmoplegic" yielded no results.

The present case RPON features are relatively characteristic when compared to the typical features of this condition. Although the literature highlights CN III as the most affected nerve in RPON (54% of cases) [3], CN VI involvement is also common in RPON (37% of cases). The absence of MRI findings such as enhancement is typical for RPON, as these are only seen in 27.6-63.3% of cases [3]. Other features of the present case that are typical for this condition include the age of onset, female sex, and photophobia [2].

Acupuncture might offer benefits in RPON by modulating pain and neuroinflammatory pathways. Previous studies suggest that acupuncture can benefit those with other forms of cranial neuropathy, particularly Bell's palsy, with evidence that acupuncture shortens its time to resolution [9-12]. In the present study, and similar cases, acupuncture point selection may be based on TCM principles, as well as be informed by protocols used for Bell's palsy and other facial pain disorders [13,14].

Chiropractic spinal manipulative therapy has also been used as a stand-alone modality or in conjunction with other therapies for Bell's palsy, with some evidence of benefit [15]. In the present case, spinal manipulation addressed cervical spine restrictions. While a direct cervicogenic mechanism of RPON has not been proposed previously, previous authors have suggested that the trigeminocervical complex becomes activated. Accordingly, it is plausible that spinal manipulation may influence the trigeminocervical complex, although this hypothesis requires further empirical investigation.

Strengths of this case report include extensive diagnostic testing and specialist visit(s), which helped arrive at the RPON diagnosis, and the extensive medical history corroborated the potential effectiveness of the treatments with a reduction in RPON episode length. As a single case report, the results may not be generalizable, and causality of treatments in symptom reduction cannot be inferred with certainty. The absence of validated outcome measures, such as sequential patient-reported outcomes or headache diaries, limits detailed insights into the response to care. The potential for synergy or individual benefit of spinal manipulation and acupuncture for RPON also remains unclear. 

## Conclusions

This case describes an adult woman with episodes of RPON that improved with chiropractic spinal manipulative therapy and acupuncture after a lack of response to pharmacologic approaches. While promising, these findings are preliminary and highlight the need for further research into nonpharmacologic approaches for this rare condition.
